# Metabolic flexibility revealed in the genome of the cyst-forming α-1 proteobacterium *Rhodospirillum centenum*

**DOI:** 10.1186/1471-2164-11-325

**Published:** 2010-05-25

**Authors:** Yih-Kuang Lu, Jeremiah Marden, Mira Han, Wesley D Swingley, Stephen D Mastrian, Sugata Roy Chowdhury, Jicheng Hao, Tamer Helmy, Sun Kim, Ahmet A Kurdoglu, Heather J Matthies, David Rollo, Paul Stothard, Robert E Blankenship, Carl E Bauer, Jeffrey W Touchman

**Affiliations:** 1 School of Life Sciences, Arizona State University, Tempe, AZ 85287, USA; 2 Indiana University, Bloomington, IN 47405, USA; 3 School of Natural Sciences, University of California - Merced, Merced, CA 95343, USA; 4 Translational Genomics Research Institute, Phoenix, AZ 85004, USA; 5 Washington University in St. Louis, St. Louis, MO 63130, USA; 6 University of Alberta, Edmonton, AB T6G 2P5, Canada

## Abstract

**Background:**

*Rhodospirillum centenum *is a photosynthetic non-sulfur purple bacterium that favors growth in an anoxygenic, photosynthetic N_2_-fixing environment. It is emerging as a genetically amenable model organism for molecular genetic analysis of cyst formation, photosynthesis, phototaxis, and cellular development. Here, we present an analysis of the genome of this bacterium.

**Results:**

*R. centenum *contains a singular circular chromosome of 4,355,548 base pairs in size harboring 4,105 genes. It has an intact Calvin cycle with two forms of Rubisco, as well as a gene encoding phosphoenolpyruvate carboxylase (PEPC) for mixotrophic CO_2 _fixation. This dual carbon-fixation system may be required for regulating internal carbon flux to facilitate bacterial nitrogen assimilation. Enzymatic reactions associated with arsenate and mercuric detoxification are rare or unique compared to other purple bacteria. Among numerous newly identified signal transduction proteins, of particular interest is a putative bacteriophytochrome that is phylogenetically distinct from a previously characterized *R. centenum *phytochrome, Ppr. Genes encoding proteins involved in chemotaxis as well as a sophisticated dual flagellar system have also been mapped.

**Conclusions:**

Remarkable metabolic versatility and a superior capability for photoautotrophic carbon assimilation is evident in *R. centenum*.

## Background

*Rhodospirillum centenum *(also known as *Rhodocista centenaria*) is a thermotolerant α-1 proteobacterium that is closely related to species of the *Azospirillum *genus [1-4]. *R. centenum *is one of the few known thermotolerant purple bacteria species. It has an optimal growth temperature of 44°C, and is capable of differentiating into metabolically dormant cysts that can survive at temperatures as high as 65°C [1-3,5]. Consequently, *R. centenum *can often be cultivated from hot springs such as those found at Yellowstone National Park [3]. *R. centenum *metabolizes a unique set of carbon sources, but is unable to utilize malate or other C_4 _dicarboxylic acids [2]. Unlike *Rhodobacter capsulatus *and other known purple non-sulfur bacteria, *R. centenum *does not repress photosystem synthesis in the presence of molecular oxygen [6].

**Figure 1:**
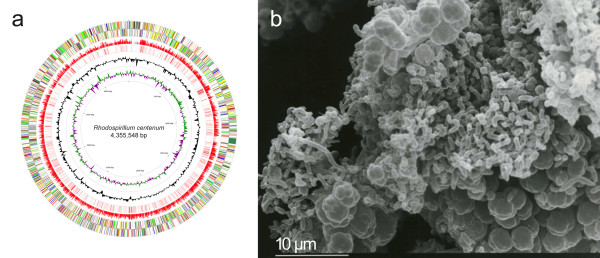
**Circular representation of the *R. centenum *chromosome**. (a) Circular representation of the *R. centenum *chromosome. The different rings represent (from outer to inner) all genes and insertion elements, color-coded by functional category (rings 1 and 2), BLASTx results (E-value = 0.0001) comparing translated *R. centenum *DNA to *R. rubrum *(NC_007643) proteins scaled according to percent identity (ring 3), BLASTn results (E-value = 0.0001) comparing *R. centenum *DNA to *R. rubrum *DNA (ring 4), deviation from average G+C content (ring 5), and deviation from average GC skew [(C-G)/(C+G); ring 6]. Color codes for gene functional categories are as follows: energy and central intermediary metabolism, green; fatty acid and phospholipid metabolism, turquoise; purine/pyrimidine/nucleoside/nucleotide biosynthesis, salmon; protein synthesis and fate, yellow; cofactor biosynthesis, pink; amino acid synthesis, orange; cellular processes and envelope, light green; DNA metabolism, red; transcription, dark blue; mobile and extra-chromosomal elements, dark green; cell division and chromosomal partitioning, light blue; general function prediction only, brown; unknown function and hypothetical proteins, dark gray. (b) Scanning electron micrograph of a *R. centenum *mature colony undergoing cyst formation, showing a heterogeneous array of vibrioid-shaped vegetative cells and clusters of spherical cyst cells.

Three morphologically distinct cell types are observed during the *R. centenum *life cycle; swim cells, swarm cells and metabolically dormant cyst cells [1,7]. Proteobacterial encystment has been reported in diverse species, and is one of several prokaryotic resting cell strategies employed for surviving environmental stress. Physiological aspects of cyst cell development have been well studied in *R. centenum*, *Azotobacter vinelandii *and *Azospirillum brasilense*, several features of which are shared by these species [4,5,8-10]. Environmental stresses, including nutrient deprivation, trigger vegetative cells to undergo a multi-stage transition into rounded, immotile cells encapsulated by complex, protective outer coats (Figure 1B). These cysts are additionally typified by the presence of large intracellular granules of the industrially significant polymer poly-hydroxybutyrate (PHB) [11]. The resulting cells have extreme desiccation resistance and also afford modest protection from stresses such as heat and UV light. While the morphological and resistive aspects of such cysts have been well studied, mechanisms that underlie the regulation of this process remain largely unknown.

Members of the cyst forming *Azospirillum *genera have significant agricultural importance. Specifically, the aerobic nitrogen fixing species *A. brasilense *and *A. lipoferum *(both closely related to *R. centenum*) are known to associate with, and stimulate the growth of, numerous grasses and cereals [12]. These bacteria may benefit such plants through their ability to aerobically fix nitrogen [12]. Interestingly, mutations that affect swim cell to swarm cell differentiation [13,14] and cyst-cell development [15] also affect plant root colonization. Thus, a better understanding of these cellular differentiation events is clearly warranted. A genome sequence advances the tools available for studies of these processes.

## Results and Discussion

### Genome Properties

*R. centenum *strain SW has a single 4,355,548-bp circular chromosome containing 4,105 genes including 4,002 open reading frames (ORFs), 12 rRNA genes, 52 tRNA genes, but no native plasmids (Table 1 and Figure 1A). The genomic G+C content of *R. centenum *(70.5%) is higher than the genome of the purple non-sulfur bacterium *Rhodospirillum rubrum *ATCC 11170 (65.4%; GenBank accession CP000230), its closest sequenced relative. Additionally, we found that 76% percent of *R. rubrum *proteins have a corresponding match in the *R. centenum *genome (e-value = 0.0001; Figure 1A). The total protein-coding content of the chromosome is 86% and the average gene length is 945 nucleotides. There are 2,633 predicted proteins that have sequence similarity to other known proteins, while 1,135 (28%) are homologous to proteins of unknown function and a further 234 (6%) are unique to *R. centenum*. The entire genome sequence has been fully annotated and a summary of genes in major functional categories is shown in Table 2. GC skew analysis failed to present a strong inflection point indicative of an origin of replication (Figure 1A). However, the replication related genes *dnaA *and *dnaN *begin at base pair positions 3,127,698 and 3,134721, respectively, which may suggest the general location of an origin.

**Table 1:** General genome characteristics

Genome size	4,355,548 bp
G+C content	70.5%
Genes	4,105
Proteins	4,002
Pseudogenes	39
Coding density	86%
Average gene length	945 nt
Structural RNAs	64
Number of CDS with a predicted function	2,633
Number of CDS similar to proteins of unknown function	1,135
Number of CDS without similarities	234
Number of insertion sequences	153

**Table 2:** Functional categories of *Rhodospirillum centenum *genes

**Gene functional category**	**No. of genes**^**1**^
Energy and central intermediary metabolisms	559
Fatty acid and phospholipid metabolism	110
Purines, pyrimidines, nucleosides, and nucleotides	70
Transport	448
Regulatory function and Signal transduction	396
Protein synthesis and fate	340
Cofactor biosynthesis	166
Amino acid synthesis	125
Cellular processes and Envelope proteins	628
DNA metabolism	78
Transcription	71
Unknown function and hypothetical proteins	1142

^1 ^Some genes are included in more than one category

Detailed metabolic pathway construction for *R. centenum *was generated from the annotated genome using the Pathway Tools suite of programs [16,17]. The predicted metabolic networks were further validated and adjusted with information from the literature that contained experimental data from taxonomically related bacteria. Based on the constructed metabolic scheme, 1,295 known enzymatic reactions involved in 263 pathways are predicted. A total of 985 putative metabolic compounds are predicted to exist in the metabolism of *R. centenum*, which appears to possess many metabolic pathways typical of purple photosynthetic bacteria in the α-proteobacteria class. Despite the existence of multiple nutrient assimilation pathways, no sulfide metabolism was identified, which verifies an earlier observation that *R. centenum *is incapable of reducing sulfide [18,19]. Nevertheless, genes required for anaerobic respiration involved in nitrate and fumarate reduction were identified.

### Carbon fixation

**Figure 2:**
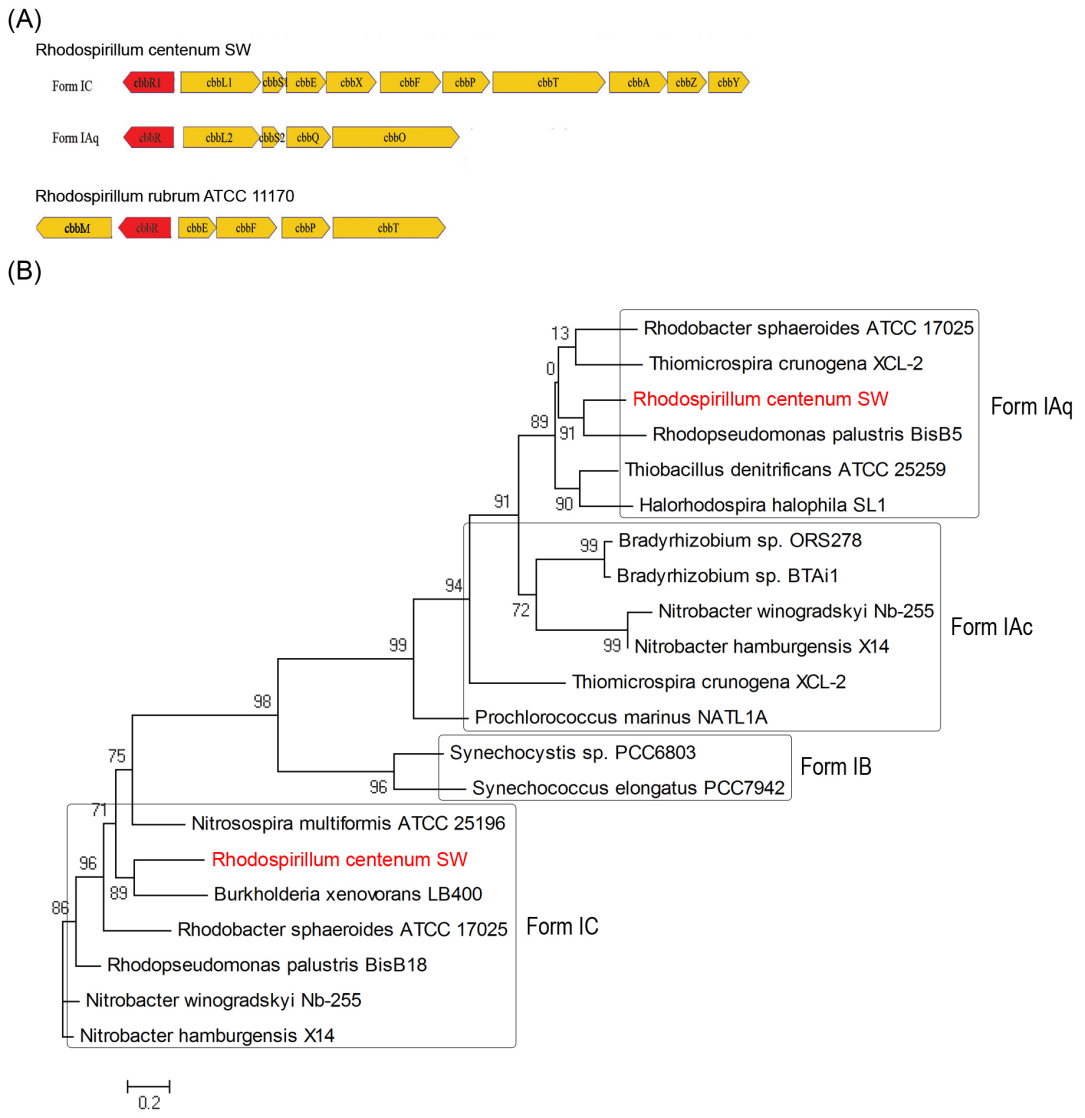
**Genomic organization and phylogenetic analysis of the two forms of Rubisco of *R. centenum***. (A) The structural gene arrangement for Form IAq and IC of Rubisco found in *R. centenum *and Form II of Rubsico in *R. rubrum *are shown. Yellow indicates those genes encoding both large and small subunits of Rubisco and the Calvin cycle enzymes. Red indicates the *cbb *operon transcriptional regulator. (B) Maximum likelihood unrooted tree of Rubisco Form IAq, IAc, IB, and IC based on a multiple protein alignment. The two forms of Rubisco found in *R. centenum *are indicated red.

The genome sequence of *R. centenum *reveals a versatile capacity for carbon fixation. Two groups of Rubisco encoding genes (tentatively named *cbbL1S1 *and *cbbL2S2*) are present within two putative operons located at distal positions of the chromosome. The first is linked with genes coding for proteins typically involved in the Calvin-Benson cycle, such as phosphoribulokinase (*prk*), whereas the second is associated with two genes (*cbbO *and *cbbQ*) that encode Rubisco activation proteins (Figure 2A). Both *cbb *operons are likely regulated by LysR-family transcription factors (CbbR1 and CbbR2), whose corresponding genes are located immediately upstream of each respective operon. Though seemingly rare, some species of bacteria have multiple forms of Rubisco [reviewed in [20]]. A phylogenetic analysis of 18 phototrophic bacteria demonstrates that *R. centenum *possesses Rubisco subtypes IAq and IC, both of which are found predominantly in proteobacteria (Figure 2B). The IC form of Rubisco is found primarily among α/β-proteobacteria while form IAq is predominantly found in chemolithotrophic β/γ-proteobacteria, with the exception of *Rhodopseudomonas palustris *BisB5.

Different kinetic properties reported for these various Rubisco forms have led to speculation that each is adapted for use in specific environmental CO_2 _concentrations, and that possession of multiple Rubisco forms may be advantageous [20]. For instance, Form IC Rubisco has a slightly lower reaction rate (*k_cat _*~2-3.2 s^-1^) than IAq (*k_cat _*~3.7 s^-1^), suggesting that they are adapted to medium-to-high and medium-to-low [CO_2_] environments, respectively [21]. The need for such extreme metabolic flexibility is reflected by the wide range of environments that non-sulfur purple bacteria inhabit. The mechanism of switching between two Rubisco forms and the roles of the dedicated regulatory proteins CbbR1 and CbbR2 are unclear in these bacteria.

Phosphoenolpyruvate carboxylase (PEPC, RC1_2446) and pyruvate orthophosphate dikinase (Pdk, RC1_1667) are present in the *R. centenum *genome. These enzymes are widespread in plants and bacteria. PEPC and Pdk (with malic enzyme) are thought to be responsible for heterotrophic carbon dioxide assimilation in *Roseobacter denitrificans *since this bacteria does not contain Rubisco [22]. However, PEPC and Pdk are also found in species that primarily use Rubisco for carbon fixation, such as *R. palustris*. Thus, the function that PEPC and Pdk perform in *R. centenum*, as well as in related Rubisco-containing purple bacteria, is unclear. An *R. palustris *PEPC-deficient strain does exhibit a slower doubling time compared with the wild-type strain grown anaerobically in the light and aerobically in the dark when pyruvate is used as a carbon source [23]. Thus, autotrophic bacteria like *R. centenum *that cannot acquire C_4 _dicarboxylic acids heterotrophically may have evolved an anaplerotic assimilation to ensure a continuous replenishment of C_4_-dicarboxylic acids needed for amino acid biosynthesis.

An analysis of the α-proteobacteria class shows that only four anoxygenic photosynthetic species are known to possess Pk, Pdk, and PEPS (phosphoenolpyruvate synthase) together (Additional File 1, Figure S1). These include two members of the *Bradyrhizobium *family, *Hoeflea phototropica*, and *R. centenum*. The others contain only Pk and Pdk, or just Pdk (e.g., the mutualistic parasites of *Rickettsiales*). The four containing Pk, Pdk, and PEPS are reported to grow poorly with pyruvate, malate, and various other dicarboxylic acids, indicating a strong dependence on carbon source such as we observe with *R. centenum *[2,24,25] .

**Figure 3:**
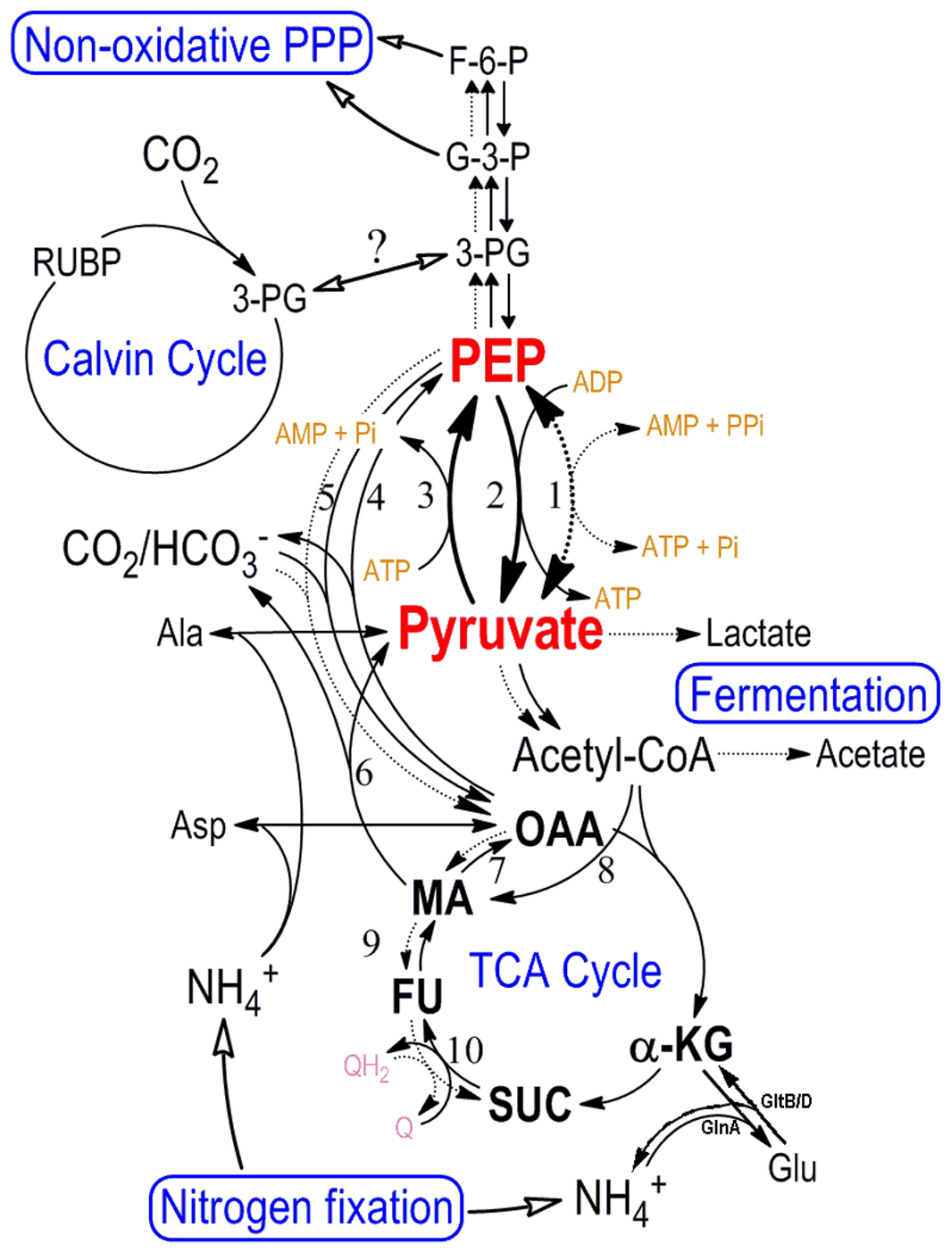
**A schematic of putative carbon flux in *R. centenum***. Aerobic reactions are designated with solid lines, micro- or anaerobic reactions with dotted lines. The participating enzymes in the major reactions are numbered: 1. pyruvate dikinase (RC1_1667); 2. pyruvate kinase (RC1_2135 and RC1_2401); 3. phosphoenolpyruvate synthase (RC1_3562); 4. phosphoenolpyruvate carboxykinase (RC1_2822); 5. phosphoenolpyruvate carboxylase (RC1_2446); 6. malic enzyme (RC1_0405 and RC1_3260); 7. malate dehydrogenase (RC1_4080); 8. malate synthase (RC1_2688); 9. fumarase (RC1_1865); 10. succinate dehydrogenase (RC1_3941). Inter-converison of fumarate and succinate (SUC) can oxidize electron carriers such as quinone (purple labeled) for anaerobic respiration. Alternatively, both pyruvate and acetyl CoA can be used in fermentation. Putatively, Glycerate-3-phosphate (3PG) produced by fixing CO_2 _into Ribulose-1,5-bipohsphate (RUBP) by Rubisco may be shuffled between the Calvin cycle and glycolysis (hollow arrowhead with question mark).

Figure 3 presents a proposed scheme in which the pyruvate/PEP interconversion driven by pyruvate kinase (Pk), pyruvate orthophosphate dikinase (Pdk), and phosphoenolpyruvate synthase (PEPS) collaborate functionally to modulate carbon flux in *R. centenum*. It is based on previous experimental data illustrating the control of pyruvate/PEP interconversion under different trophic conditions in *R. denitrificans*, *R. rubrum*, and Archaea [22,26,27] . When aerobic respiration is suppressed due to the lack of oxygen, Pk is functionally replaced by Pdk that continuously supplies pyruvate [26,27]. We speculate that PEPS collaborates with both Pk and Pdk under nitrogen-fixing conditions, where it supplies a stable supplement of dicarboxylic acids through an internal pyruvate pool for amino acid biosynthesis. PEPS-driven gluconeogenesis in *R. centenum *may contribute to the balance of carbon flux for the non-oxidative pentose phosphate pathway when the rate of CO_2 _fixation is limited.

### Metabolism

*R. centenum *is an active nitrogen fixer. The *nif *genes of *R. centenum *are located in two distant regions: the first region consists of 22 genes that essentially include *nifIXENKDHTZBAVW *for nitrogenase biosynthesis, *modBC *for molybdenum transport, and *fixABCX *for electron transport. Phylogenetic analysis using a concatenated alignment of the nitrogenase structural genes *nifHDK *shows that *R. centenum *falls within a clade containing *R. sphaeroides*, *R. rubrum *and several *Rhizobiales *(not shown). This result suggests that this group of nitrogenases originated from a common ancestor.

Neither iron nor vanadium nitrogenases, coded by *anf *and *vnfVHDK*, respectively, were identified in the *R. centenum *genome. In *R. rubrum *the regulatory enzymes dinitrogenase reductase ADP-ribosyl transferase (DRAT) and dinitrogenase reductase-activating glycohydrolase (DRAG) regulate nitrogenase activity by reversible ADP-ribosylation of NifH [28,29]. An absence of these genes in *R. centenum *suggests that these organisms have different environmental requirements for nitrogen fixation. While *R. centenum *possesses a nitrogenase for nitrogen fixation, utilization of inorganic nitrogen compounds as an alternative nitrogen source is restricted. Unlike *R. denitrificans*, which has a large complement of genes for nitrogen metabolism (but not nitrogenase), the *R. centenum *genome does not contain genes which encode an assimilatory nitrite reductase (*nirSCFDGHJN*) for the denitrification pathway. Yet, the nitrate reduction by periplasmic nitrate reductase (*napABCDEF*) seems to be intact.

We identified the presence of two cytosolic detoxifying enzymes, arsenate and mercuric reductase. To our knowledge, the latter has never been reported in a purple bacterium. Two copies of arsenate reductase, encoded by *arsC1 *and *arsC2*, are present (RC1_2995 and RC1_3700). One copy is associated with genes encoding an arsenate efflux pump (*arc3*) and an arsenic resistance repressor (*arsR*). In both ArsC proteins, the cysteine residues that presumably bind arsenate are conserved, but the protein sequences share less than 20% overall identity. The presence of *arc3 *and *arsR *implies that arsenate reductase is an inducible enzyme when arsenate is present. There is one copy of mercuric reductase encoding *merA *(RC1_2279). Generally, these two detoxifying systems are energy dependent, with arsenate reductase using either thioredoxin or glutaredoxin while mercuric reductase uses NADPH [30].

### Cyst formation

To date, the only concerted effort at finding regulators of encystment (Figure 1B) in any bacterial species has been undertaken in *R. centenum*, where a screen for Tn5 mutants displaying de-repressed encystment on nutrient-rich media uncovered several such components [31]. A subset of identified elements lay within an operon of chemotaxis-like genes (*che3)*, individual in-frame deletions of which exhibit opposing premature 'hyper-cyst' or delayed 'hypo-cyst' phenotypes [32]. Predicted signaling components of note in this cluster include a small receiver domain protein (CheY_3_; RC1_2133), a hybrid sensor kinase-receiver CheA homolog (CheA_3_; RC1_2127) and a similarly hybridized kinase-receiver protein (CstS_3_; RC1_2124) that also contains a PAS sensory domain. As ultimate control over cyst cell development no doubt occurs at the transcriptional level, the immediate output of this system is unclear, as none of the aforementioned components has an obvious DNA binding domain. Whether or not Che_3 _signaling ultimately regulates timing of encystment directly, through the phosphorylation of a classic response regulator, or does so by means of a more indirect mechanism remains to be elucidated. This screen also identified two genomically orphaned, cytoplasmic sensor kinases (CstS1, RC1_2847 and CstS2, RC1_2047), both predicted to contain PAS and PAC sensory motifs with CstS1 also containing a GAF and a receiver domain [31]. Deletion of *cstS1 *and *cstS2 *have contrasting respective hypo-cyst and hyper-cyst phenotypes, and epistatic analyses of these genes and *che_3 _*components indicate a complex signaling hierarchy into which contributions are undoubtedly made by hitherto unknown regulatory elements (Berleman and Bauer, Unpublished Data). In fact further screens in our lab have uncovered several such regulators, including two sensor kinases, RC1_0896 and RC1_3465 (Marden and Bauer, Unpublished Data). These genes were independently identified by a similar screen in a separate lab where they are currently the subject of genetic characterization (Bird, Manuscript in preparation).

### Photosynthetic and chlorophyll biosynthetic proteins

**Figure 4:**
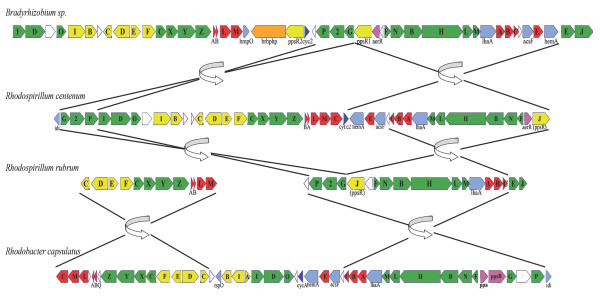
**Photosynthesis gene cluster arrangement in the purple bacteria *Bradyrhizobium sp*, *R. centenum*, *R. rubrum*, and *Rhodobacter capsulatus***. Genes are presented as arrows indicating their direction of transcription. Genes are colored as listed: chlorophyll biosynthesis (*bch*) in green, carotenoid biosynthesis (*crt*) in yellow, reaction centers (*puf*) and light-harvesting complexes (*puh*) in red, regulatory proteins (*aerR*, *ppsR*, and *crtJ*) in purple, other functional proteins in light blue, and uncharacterized genes in white. Brbphp in orange is a bacteriophytochrome unique in *Bradyrhizobium sp*. Lines illustrate gene rearrangement, and arrows illustrate the inversion of large superoperonal clusters.

Despite striking dissimilarity in the genomic organization of photosynthesis genes in different photosynthetic species, most of the genes that carry out bacterial chlorophyll and carotenoid biosynthesis in *R. centenum *are found in a single photosynthetic gene cluster (PGC; Figure 4). The photosynthesis genes are organized into seven major operons. The gene cluster *hemA-puhH-acsF-puhCBA-bch-lhaAb-chLMHBNF *is located immediately downstream of *pufMLAB-bchZYXC-crtFEDCBI *in *R. centenum*. This is in contrast to *Bradyrhizobium *sp. where it maps immediately upstream of *aerR-ppsR1-bchG2P*. The carotenoid biosynthesis gene *crtA *found in *Rhodobacter capsulatus *(among others) is not found in *R. centenum *(or *Bradyrhizobium *sp). The bch/heme biosynthesis genes *acsFI, puhE*, *hemA*, and the *cyc2 *gene encoding cytochrome *c*_2_, are present in the genome. Thus, the overall organization of the *R. centenum *PGC is similar to the PGC of *Bradyrhizobium *sp. but not closely related to that of *Rhodobacter *species. We also found that *R. centenum *and *R. rubrum *do not share contiguity of their PGCs, where the *R. rubrum *PGC is separated into two clusters in distant regions of the chromosome.

### Photoreceptors

The *R. centenum ppr *gene represents the first bacteriophytochrome identified in purple bacteria [33]. Distinctive characteristics of Ppr, with respect to other bacteriophytochromes, have been discussed in detail elsewhere [reviewed in [34]]. In addition to *ppr*, a second gene (RC1_3803) is predicted to encode an additional bacteriophytochrome. RC1_3803 does not possess a photoactive yellow protein (PYP) domain, and has 45% and 55% similarity to both the photosensory core domain (PCD) and histidine kinase domain (HKD) of Ppr, respectively (not shown). A search of public protein databases identified a number of bacteriophytochromes that show homology to RC1_3803 (not shown). Based on characteristics of other bacteriophytochromes, we hypothesize that RC1_3803 may absorb near far-red light, as that wavelength of light is reported to promote negative phototaxis of *R. centenum *and is in the region of the spectrum where other bacteriophytochromes exhibit spectral absorbance [7], reviewed in [34].

Finally, there are two genes coding for flavin-binding photoreceptors. One gene (RC1_2193) putatively codes for a small blue light photoreceptor utilizing a flavin (BLUF) and a second protein (RC1_0351) putatively encodes a histidine kinase containing a light-oxygen-voltage (LOV) domain. Both of these putative photoreceptors likely utilize FAD or FMN as a chromophore to absorb blue light to promote a conformational change to elicit an output response. Neither protein has been genetically disrupted, but they may play a role in controlling light regulated physiology or behavior in *R. centenum*.

### Flagella

**Figure 5:**
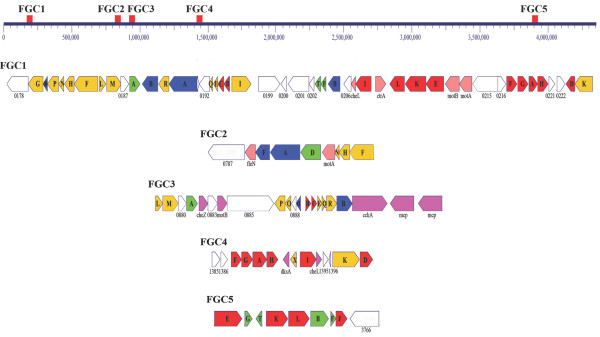
**Flagellar gene cluster (FGC) arrangement**. A linear representation of the *R. centenum *genome with the relative positions of the five flagellar gene clusters is shown at the top. Beneath, genes in individual clusters are presented as arrows indicating the direction of transcription and colored as follows: *flg *genes, red; *fli *genes, yellow; *fla *and *flb *genes, blue; *flh *genes, green; regulatory and other functional proteins, pink; and uncharacterized genes, white.

*R. centenum *synthesizes two flagella, a constitutive polar flagellum for swimming motility and inducible lateral flagella required for swarming motility on viscous or solid media [35]. We identified 72 flagella genes in the *R. centenum *genome distributed among five major flagellar gene clusters (FGCs) at various regions along the chromosome (Figure 5). Most structural genes are duplicated while *flgC*, *flgF*, *flhF*, *fliL*, and *fliN *have either three or four copies each. Several genes involved in regulation, export or assembly (*flhF*, *fliO*, *fliX*, *flaA*, *flaG*, *flbD*, and *fleN) *are present as a single copy.

**Figure 6:**
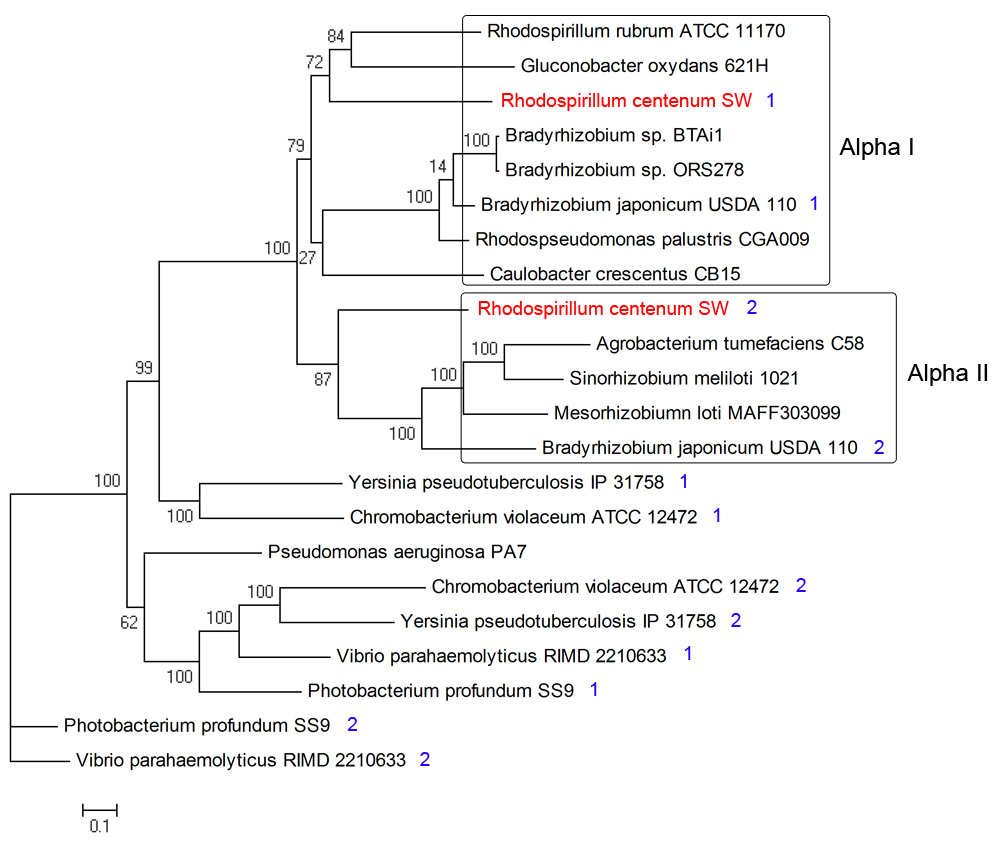
**Maximum likelihood tree based on conserved flagellar proteins depicting the position of *R. centenum *flagellar systems among other flagellated bacteria**. The tree is based on a concatenated alignment of 11 flagellar proteins (*Flg*) that are present in all primary and secondary flagellar systems [36]. A blue "1" or "2" after the species name indicates primary and secondary flagella systems, respectively. Alpha I (alphaproteobacteria group I) and Alpha II (alphaproteobacteria group II) are boxed. The primary (polar) and secondary (lateral) flagella of *R. centenum *are in red.

Lateral and polar flagellar systems have diverged twice in α-proteobacteria as well as in the common ancestor of β/γ-proteobacteria [36]. Many of the duplicated *fla *genes in *R. centenum *exhibit poor sequence similarity to their reciprocal pair indicating a high degree of diversity among the lateral and polar flagella genes (Additional File 1, Table S1). It also does not rule out that either the polar or lateral flagella genes may have been derived by lateral gene transfer. A phylogenetic tree using a concatenated alignment of eleven *flg *genes from *R. centenum *indicates that the lateral flagellar system of *R. centenum *indeed has a distinct origin from the polar flagellar system (Figure 6). The four small clusters (FGC2, FGC3, FGC4 and FGC5) that map to different positions on the chromosome have subunits predicted to constitute components of the polar flagellum. Structural components of the lateral flagella are predicted to reside among the large FGC1 cluster.

We have obtained several mutations in the transcription factor FlbD that disrupt synthesis of the polar, but not lateral flagella (D. Rollo and C. Bauer, unpublished results). These results indicate that expression of the polar and lateral flagella genes are distinct. Also, several insertion mutations that affect synthesis of both the lateral and polar flagellum map to components of the type III export system comprised of FliI, FlhA, FlhB and FliL, indicating that this export apparatus is used by both flagella systems.

There are 13 additional genes that include three functionally identified, four functionally predicted, and six of unknown function distributed among the *fla *clusters. These include genes encoding a CheY-like receiver protein (RC1_0209), lytic muramyl transglycoylase (RC1_0192), DNA polymerase III (RC1_0787), PPE-repeat proteins (RC1_0178), tetratricopeptide TPR_2 (RC1_0215), DNA binding protein (RC1_0222), and an ATPase involved in DNA repair (RC1_0187).

### Chemotaxis and Signal Transduction

The three previously identified operons (Che1, Che2 and Che3) encoding chemotaxis-like proteins were confirmed to represent the entirety of such 'Che-like' clusters in *R. centenum *[32,37,38] . Each contains homologs of the *E. coli *chemotactic proteins CheA, CheY, CheW, CheB and CheR, with an additional CheW in the Che3 cluster (CheW3a and CheW3b) and a tripartate CheW in the Che2 cluster composed of three distinct CheW domains. Atypical CheA sensor kinases are also present in the Che1 and Che2 clusters, as each is hybridized to a receiver domain. Also of note are genes encoding non-canonical chemotactic components, two small proteins of unknown function in the Che2 cluster (RC1_0336 and RC1_0341) and an additional sensor kinase receiver domain hybrid (CstS3; RC1_2124) in the Che3 cluster.

The functions of all three chemotaxis operons have been elucidated. Both chemotactic and phototactic behavior in *R. centenum *are under Che1 control, as strains with Che1 component disruptions have motility phenotypes similar to those of *E. coli *chemotactic mutants [39]. The Che2 cluster is involved in lateral flagella biosynthesis, as strains deleted of Che2 components are either hyper-flagellated or lack flagella completely, but remain chemotactic [32]. Lastly, the Che3 cluster directs timing of encystment, as deletions of Che3 components produce strains that are either early or delayed in cyst cell development [37]. The molecular mechanisms by which the latter two clusters achieve such altered functions are yet to be elucidated.

Whereas CheA, CheW, CheB and CheR homologs were only identified within these chemotaxis clusters (a CheB-CheR hybrid, RC1_3878, was discovered), 19 genes encode for small, stand-alone CheY-like receiver domain proteins. Besides the three Che cluster-associated CheY encoding homologs, two of these *cheY*-like genes are associated with neighboring genes encoding chemosensory proteins. The first (RC1_0955) is adjacent to one of two putative CheZ phosphatases (RC1_0954 and RC1_0882). The second (RC1_0353) is part of a potential operon with genes encoding a small hypothetical protein (RC1_0352), a methyl accepting chemotaxis domain protein (MCP; RC1_0354) and a homolog of the response regulator *fixJ *(RC1_0355). Whether these stand-alone receiver domains play a part in chemotactic signal transduction will require significant genetic and biochemical characterization.

A total of 33 genes encoding MCP domains were identified in our analysis, only three of which have been previously genetically characterized. The first identified, Ptr, was shown to be responsible for photosensory perception in *R. centenum *[40]. MCP2 and MCP3, which are respectively and functionally associated with the aforementioned Che2 and Che3 clusters, have functions independent of taxis, since gene deletion strains are chemotactic but instead are either hyper-flagellated (Δ*mcp2*) or delayed in encystment (Δ*mcp3*) [32,37]. As discussed above, a methyl-accepting sensory transducer is also tightly linked with a homolog of *fixJ*, which is known to be a transcription activator for nitrogen fixation in microaerobic conditions [41]. We speculate that this MCP may thus have a role in altering bacterial swimming to approach the microaerobic conditions that are optimal for nitrogen fixation. The relatively large number of new and uncharacterized MCP domain-containing proteins suggests a capacity to sense and respond to a wide variety of extracellular signals, either with a tactic response, or with the alternate functions controlled by the Che2 and Che3 pathways.

In our analysis of two-component signal transduction, 55 identified genes are predicted to encode sensor kinases, 16 of which are hybridized to receiver domains. Excluding the latter group, 54 proteins are predicted to contain receiver domains. Of these 19 are stand-alone receiver domains with the remainder fused to assorted sensory and/or output domains. Lastly, 8 predicted proteins contain a histidine-containing phosphotransfer domain; three Class II histidine kinases (all *R. centenum *CheA proteins), two within hybrid Class I histidine kinases (RC1_0633 and RC1_2262), two stand-alone domains (RC1_2126 and RC1_3033) and one associated with a receiver domain (RC1_1779).

## Conclusions

Analysis of the *R. centenum *genome demonstrates that both Rubisco- and PEPC- derived carbon assimilation can compensate for the inability to utilize malate or other C_4 _dicarboxylic acids in the *R. centenum *environment. Many newly identified genes that are discussed in this report have advanced our knowledge of the structure and origin of the *R. centenum *PGC, the complex life cycle involving differentiation from swim to swarm cells, and the differentiation into heat and desiccation resistant resting cysts. *R. centenum *also contains many sensory proteins such as bacteriophytochromes that control gene expression in response to complex environmental stimuli.

The completion of the *R. centenum *genome impacts the study of cyst cell development in particular, already allowing the identification of an *A. brasilense flcA *homolog. FlcA is a transcriptional regulator of *A. brasilense *encystment, and appears to have a role in *R. centenum *encystment (Marden and Bauer, Unpublished Data). The sequenced genome has also allowed for the discovery of an additional sensor kinase involved in encystment, RC1_2747. This gene was originally disrupted and identified in a hyper-cyst screen, however the transposition occurred in a region with low sequence similarity and was placed in a class of unknown genes [5].

*R. centenum *is emerging as a model organism for molecular genetic analysis of cyst formation, photosynthesis, phototaxis, and cellular development. This species is genetically amenable, with a variety of genetic tools already developed to explore these processes. The generation of a complete and annotated genome sequence establishes the genetic infrastructure for such studies, provides a framework to organize all the genetic information about the organism, and catalyzes future 'omics' research.

## Methods

*R. centenum *strain SW (ATCC 51521) originated from hot spring mud in Wyoming, United States. A single colony was grown anaerobically and total DNA was isolated using proteinase K treatment followed by phenol extraction. The DNA was fragmented by kinetic shearing, and three shotgun libraries were generated: small and medium insert libraries in the plasmid pOTWI3 (using size fractions of 2-3 kb and 6-8 kb, respectively), and a large insert fosmid library in pEpiFOS-5 (insert sizes ranging from 28-47 kb), which was used as a scaffold. The relative amount of sequence coverage obtained from the small, medium, and large insert libraries was approximately 8×, 1×, and 1×, respectively. The whole genome sequence was established from 55,014 end sequences (giving 9.7× coverage) derived from these libraries using dye terminator chemistry on ABI 3730xl automated sequencers. The sequence was assembled with the program Arachne [42] and finished as described previously [43]. The complete and annotated genome sequence of *R. centenum *has been deposited at DDBJ/EMBL/GenBank under the accession number CP000613.

Initial automated annotation of the genome was performed with the TIGR/JCVI Annotation Engine http://www.tigr.org/AnnotationEngine, where it was processed by TIGR's prokaryotic annotation pipeline. Included in the pipeline is gene finding with Glimmer, Blast-extend-repraze (BER) searches, HMM searches, TMHMM searches, SignalP predictions, and automatic annotations from AutoAnnotate. The manual annotation tool Manatee (manatee.sourceforge.net) was used to carefully review and confirm the annotation of every gene. Pseudogenes contained one or more mutations that would ablate expression; each inactivating mutation was subsequently checked against the original sequencing data. The circular genome map was created using the program CGView [44].

Mulitple amino acid sequence alignment and phylogenetic trees for this study were built using Muscle [45], Gblocks [46], PhyML [47], and MEGA 4.0 [48] as previously described [36]. Some of the sequences used in our analysis were collected from the JGI Integrated Microbial Genomes browser http://img.jgi.doe.gov/cgi-bin/pub/main.cgi. The Pathway-Tools software was employed for predicting and comparing metabolic pathways of *R. centenum *[16,17]. The initial process of metabolic construction for *R. centenum *was automatic and involved building each pathway based on genome annotation results and the presence of each pathway in the MetaCyc database [17]. A further step to validate the accuracy of the constructed metabolic network was carried out based on supporting information from the scientific literature.

### Electron microscopy

*R. centenum *cultures were harvested and washed three times in phosphate buffer and then pipetted onto CENBA plates in 5 μl aliquots. After 1, 2 and 3 days incubation, the cell spots were harvested, fixed in 5% glutaraldehyde/100 mM HEPES/2 mM MgCl_2 _and analyzed by transmission electron microscopy as described previously [18]. Mature *R. centenum *colonies were analyzed by scanning electron microscopy, performed as described previously [49].

## Authors' contributions

REB, CEB, and JWT designed research; MH, JM, SDM, PS and SRC conducted experiments and contributed analytic tools; Y-KL, MH, JM, WDS, JH, TH, SK, AAK, HJM, DR, REB, CEB and JWT analyzed data; and Y-KL, JM, CEB and JWT wrote the paper.

## Supplementary Material

Additional file 1 **Neighbor-joining 16S rDNA phylogeny of the alpha-proteobacteria class indicating the distribution of Pk, Pdk, and PEPS**. A phylogenetic analysis of alpha-proteobacteria taxa that are annotated further to indicate phototrophism and the presence (or absence) of genes for Rubisco, Pk, Pdk, and PEPS. Characterization of R. centenum flagella genes. A table describing the gene name, copy number, similarity, and predicted function of all *R. centenum *flagella-associated genes. Click here for file

## References

[B1] Favinger J Stadtwald R Gest H *Rhodospirillum centenum*, sp. nov., a thermotolerant cyst-forming anoxygenic photosynthetic bacterium Antonie van Leeuwenhoek 1989 11 3 291 296 10.1007/BF00393857 2757370

[B2] Stadtwald-Demchick R Turner FR Gest H Physiological properties of the thermotolerant photosynthetic bacterium, *Rhodospirillum centenum* FEMS Microbiol Lett 1990 11 139 144 10.1111/j.1574-6968.1990.tb13851.x

[B3] Nickens D Fry CJ Ragatz LR Bauer CE Gest H Biotype of the nonsulfur purple photosynthetic bacterium, *Rhodospirillum centenum* Arch Microbiol 1996 11 91 96 10.1007/s002030050302 7710317

[B4] Stoffels M Castellanos T Hartmann A Design and application of new 16S rRNA-targeted oligonucleotide probes for the *Azospirillum-Skermanella-Rhodocista*-cluster Syst Appl Microbiol 2001 11 1 83 97 10.1078/0723-2020-00011 11403403

[B5] Berleman JE Bauer CE Characterization of cyst cell formation in the purple photosynthetic bacterium *Rhodospirillum centenum* Microbiology 2004 11 383 390 10.1099/mic.0.26846-0 14766916

[B6] Yildiz FH Gest H Bauer CE Attenuated effect of oxygen on photopigment synthesis in *Rhodospirillum centenum* J Bacteriol 1991 11 17 5502 5506 1885527 10.1128/jb.173.17.5502-5506.1991 PMC208263

[B7] Ragatz L Jiang ZY Bauer CE Gest H Macroscopic phototactic behavior of the purple photosynthetic bacterium *Rhodospirillum centenum* Arch Microbiol 1995 11 1 1 6 10.1007/BF00262196 7710317

[B8] Fani R Bandi C Bazzicalupo M Ceccherini MT Fancelli S Gallori E Gerace L Grifoni A Miclaus N Damiani G Phylogeny of the genus *Azospirillum *based on 16S rDNA sequence FEMS Microbiol Lett 1995 11 2-3 195 200 7607400 10.1111/j.1574-6968.1995.tb07579.x

[B9] Sadasivan L Neyra CA Cyst production and brown pigment formation in aging cultures of *Azospirillum brasilense *ATCC 29145 J Bacteriol 1987 11 4 1670 1677 3104311 10.1128/jb.169.4.1670-1677.1987 PMC211998

[B10] Stevenson LH Socolofsky MD Cyst formation and poly-beta-hydroxybutyric acid accumulation in *Azotobacter* J Bacteriol 1966 11 1 304 310 5903098 10.1128/jb.91.1.304-310.1966 PMC315949

[B11] Anderson AJ Dawes EA Occurrence, metabolism, metabolic role, and industrial uses of bacterial polyhydroxyalkanoates Microbiol Reviews 1990 11 4 450 472 10.1128/mr.54.4.450-472.1990 PMC372789 2087222

[B12] Steenhoudt O Vanderleyden J *Azospirillum*, a free-living nitrogen-fixing bacterium closely associated with grasses: genetic, biochemical and ecological aspects FEMS Microbiol Rev 2000 11 4 487 506 10.1111/j.1574-6976.2000.tb00552.x 10978548

[B13] Moens S Schloter M Vanderleyden J Expression of the structural gene, laf1, encoding the flagellin of the lateral flagella in *Azospirillum brasilense *Sp7 J Bacteriol 1996 11 16 5017 5019 8759869 10.1128/jb.178.16.5017-5019.1996 PMC178288

[B14] Moens S Vanderleyden J Functions of bacterial flagella Crit Rev Microbiol 1996 11 2 67 100 10.3109/10408419609106456 8817078

[B15] Pereg Gerk L Gilchrist K Kennedy IR Mutants with enhanced nitrogenase activity in hydroponic *Azospirillum brasilense*-wheat associations Appl Environ Microbiol 2000 11 5 2175 2184 10.1128/AEM.66.5.2175-2184.2000 10788397 PMC101470

[B16] Karp PD Paley S Romero P The Pathway Tools software Bioinformatics 2002 11 1 S225 232 12169551 10.1093/bioinformatics/18.suppl_1.s225

[B17] Caspi R Foerster H Fulcher CA Kaipa P Krummenacker M Latendresse M Paley S Rhee SY Shearer AG Tissier C Walk TC Zhang P Karp PD The MetaCyc Database of metabolic pathways and enzymes and the BioCyc collection of Pathway/Genome Databases Nucleic Acids Res 2008 36 Database D623 631 17965431 10.1093/nar/gkm900 PMC2238876

[B18] Favinger J Stadtwald R Gest H *Rhodospirillum centenum*, sp. nov., a thermotolerant cyst-forming anoxygenic photosynthetic bacterium Antonie Van Leeuwenhoek 1989 11 3 291 296 10.1007/BF00393857 2757370

[B19] Nickens D Fry CJ Ragatz LR Bauer CE Gest H Biotype of the nonsulfur purple photosynthetic bacterium, *Rhodospirillum centenum* Arch Microbiol 1996 11 91 96 10.1007/s002030050302 7710317

[B20] Badger MR Bek EJ Multiple Rubisco forms in proteobacteria: their functional significance in relation to CO2 acquisition by the CBB cycle J Exp Bot 2008 11 7 1525 1541 10.1093/jxb/erm297 18245799

[B21] Tabita FR Microbial ribulose 1,5-bisphosphate carboxylase/oxygenase: a different perspective Photosynthesis Res 1999 11 1 28 10.1023/A:1006211417981

[B22] Tang KH Feng X Tang YJ Blankenship RE Carbohydrate metabolism and carbon fixation in *Roseobacter denitrificans *OCh114 PLoS One 2009 11 10 e7233 10.1371/journal.pone.0007233 19794911 PMC2749216

[B23] Inui M Dumay V Zahn K Yamagata H Yukawa H Structural and functional analysis of the phosphoenolpyruvate carboxylase gene from the purple nonsulfur bacterium *Rhodopseudomonas palustris *No. 7 J Bacteriol 1997 11 15 4942 4945 9244286 10.1128/jb.179.15.4942-4945.1997 PMC179345

[B24] Biebl H Tindall BJ Pukall R Lunsdorf H Allgaier M Wagner-Dobler I *Hoeflea phototrophica *sp. nov., a novel marine aerobic alphaproteobacterium that forms bacteriochlorophyll a Int J Syst Evol Microbiol 2006 11 Pt 4 821 826 10.1099/ijs.0.63958-0 16585702

[B25] Fleischman D Kramer D Photosynthetic rhizobia Biochim Biophys Acta 1998 11 1 17 36 10.1016/S0005-2728(98)00011-5 9554937

[B26] Tjaden B Plagens A Dorr C Siebers B Hensel R Phosphoenolpyruvate synthetase and pyruvate, phosphate dikinase of Thermoproteus tenax: key pieces in the puzzle of archaeal carbohydrate metabolism Mol Microbiol 2006 11 2 287 298 10.1111/j.1365-2958.2006.05098.x 16573681

[B27] Grammel H Gilles ED Ghosh R Microaerophilic cooperation of reductive and oxidative pathways allows maximal photosynthetic membrane biosynthesis in *Rhodospirillum rubrum* Appl Environ Microbiol 2003 11 11 6577 6586 10.1128/AEM.69.11.6577-6586.2003 14602616 PMC262267

[B28] Schneider K Gollan U Selsemeier-Voigt S Plass W Muller A Rapid purification of the protein components of a highly active "iron only" nitrogenase Naturwissenschaften 1994 11 9 405 408 10.1007/BF01132694 7969501

[B29] Wang H Noren A Metabolic regulation of nitrogen fixation in *Rhodospirillum rubrum* Biochem Soc Trans 2006 11 Pt 1 160 161 16417510 10.1042/BST0340160

[B30] Silver S Phung le T A bacterial view of the periodic table: genes and proteins for toxic inorganic ions J Ind Microbiol Biotechnol 2005 11 11-12 587 605 10.1007/s10295-005-0019-6 16133099

[B31] Berleman JE Hasselbring BM Bauer CE Hypercyst mutants in *Rhodospirillum centenum *identify regulatory loci involved in cyst cell differentiation J Bacteriol 2004 11 17 5834 5841 10.1128/JB.186.17.5834-5841.2004 15317789 PMC516826

[B32] Berleman JE Bauer CE A che-like signal transduction cascade involved in controlling flagella biosynthesis in *Rhodospirillum centenum* Mol Microbiol 2005 11 5 1390 1402 10.1111/j.1365-2958.2005.04489.x 15720548

[B33] Jiang Z Swem LR Rushing BG Devanathan S Tollin G Bauer CE Bacterial photoreceptor with similarity to photoactive yellow protein and plant phytochromes Science 1999 11 5426 406 409 10.1126/science.285.5426.406 10411503

[B34] Giraud E Vermeglio A Bacteriophytochromes in anoxygenic photosynthetic bacteria Photosynthesis Res 2008 11 2 141 153 10.1007/s11120-008-9323-0 18612842

[B35] McClain J Rollo DR Rushing BG Bauer CE *Rhodospirillum centenum *utilizes separate motor and switch components to control lateral and polar flagellum rotation J Bacteriol 2002 11 9 2429 2438 10.1128/JB.184.9.2429-2438.2002 11948156 PMC134980

[B36] Liu R Ochman H Origins of flagellar gene operons and secondary flagellar systems J Bacteriol 2007 11 19 7098 7104 10.1128/JB.00643-07 17644605 PMC2045201

[B37] Berleman JE Bauer CE Involvement of a Che-like signal transduction cascade in regulating cyst cell development in *Rhodospirillum centenum* Mol Microbiol 2005 11 6 1457 1466 15916598 10.1111/j.1365-2958.2005.04646.x

[B38] Jiang ZY Bauer CE Analysis of a chemotaxis operon from *Rhodospirillum centenum* J Bacteriol 1997 11 18 5712 5719 9294426 10.1128/jb.179.18.5712-5719.1997 PMC179458

[B39] Jiang ZY Gest H Bauer CE Chemosensory and photosensory perception in purple photosynthetic bacteria utilize common signal transduction components J Bacteriol 1997 11 18 5720 5727 9294427 10.1128/jb.179.18.5720-5727.1997 PMC179459

[B40] Jiang ZY Bauer CE Component of the *Rhodospirillum centenum *photosensory apparatus with structural and functional similarity to methyl-accepting chemotaxis protein chemoreceptors J Bacteriol 2001 11 1 171 177 10.1128/JB.183.1.171-177.2001 11114914 PMC94863

[B41] Ton-Hoang B Salhi M Schumacher J Da Re S Kahn D Promoter-specific involvement of the FixJ receiver domain in transcriptional activation J Mol Biol 2001 11 4 583 589 10.1006/jmbi.2001.5014 11575915

[B42] Batzoglou S Jaffe DB Stanley K Butler J Gnerre S Mauceli E Berger B Mesirov JP Lander ES ARACHNE: a whole-genome shotgun assembler Genome Res 2002 11 1 177 189 10.1101/gr.208902 11779843 PMC155255

[B43] Swingley WD Sadekar S Mastrian SD Matthies HJ Hao J Ramos H Acharya CR Conrad AL Taylor HL Dejesa LC Shah MK O'Huallachain ME Lince MT Blankenship RE Beatty JT Touchman JW The complete genome sequence of *Roseobacter denitrificans *reveals a mixotrophic rather than photosynthetic metabolism J Bacteriol 2007 11 3 683 690 10.1128/JB.01390-06 17098896 PMC1797316

[B44] Stothard P Wishart DS Circular genome visualization and exploration using CGView Bioinformatics 2005 11 4 537 539 10.1093/bioinformatics/bti054 15479716

[B45] Edgar RC MUSCLE: multiple sequence alignment with high accuracy and high throughput Nucleic Acids Res 2004 11 5 1792 1797 10.1093/nar/gkh340 15034147 PMC390337

[B46] Castresana J Selection of conserved blocks from multiple alignments for their use in phylogenetic analysis Mol Biol Evol 2000 11 4 540 552 10742046 10.1093/oxfordjournals.molbev.a026334

[B47] Guindon S Gascuel O A simple, fast, and accurate algorithm to estimate large phylogenies by maximum likelihood Systematic Biol 2003 11 5 696 704 10.1080/10635150390235520 14530136

[B48] Tamura K Dudley J Nei M Kumar S MEGA4: Molecular Evolutionary Genetics Analysis (MEGA) software version 4.0 Mol Biol Evol 2007 11 8 1596 1599 10.1093/molbev/msm092 17488738

[B49] Nickens D Fry C Ragatz L Bauer CE Gest H Biotype of the purple nonsulfur photosynthetic bacterium, *Rhodospirillum centenum* Arch Microbiol 1996 11 91 96 10.1007/s002030050302 7710317

