# Supplementary material for: Metabolic flexibility revealed in the genome of the cyst-forming α-1 proteobacterium Rhodospirillum centenum
Source: BMC Genomics. 2010 May 25;11:325. doi: 10.1186/1471-2164-11-325 (PMC2890560; doi:10.1186/1471-2164-11-325)
Supplement: Additional file 1 — Neighbor-joining 16S rDNA phylogeny of the alpha-proteobacteria class indicating the distribution of Pk, Pdk, and PEPS. A phylogenetic analysis of alpha-proteobacteria taxa that are annotated further to indicate phototrophism and the presence (or absence) of genes for Rubisco, Pk, Pdk, and PEPS. Characterization of R. centenum flagella genes. A table describing the gene name, copy number, similarity, and predicted function of all R. centenum flagella-associated genes. [file 1471-2164-11-325-S1.PDF]

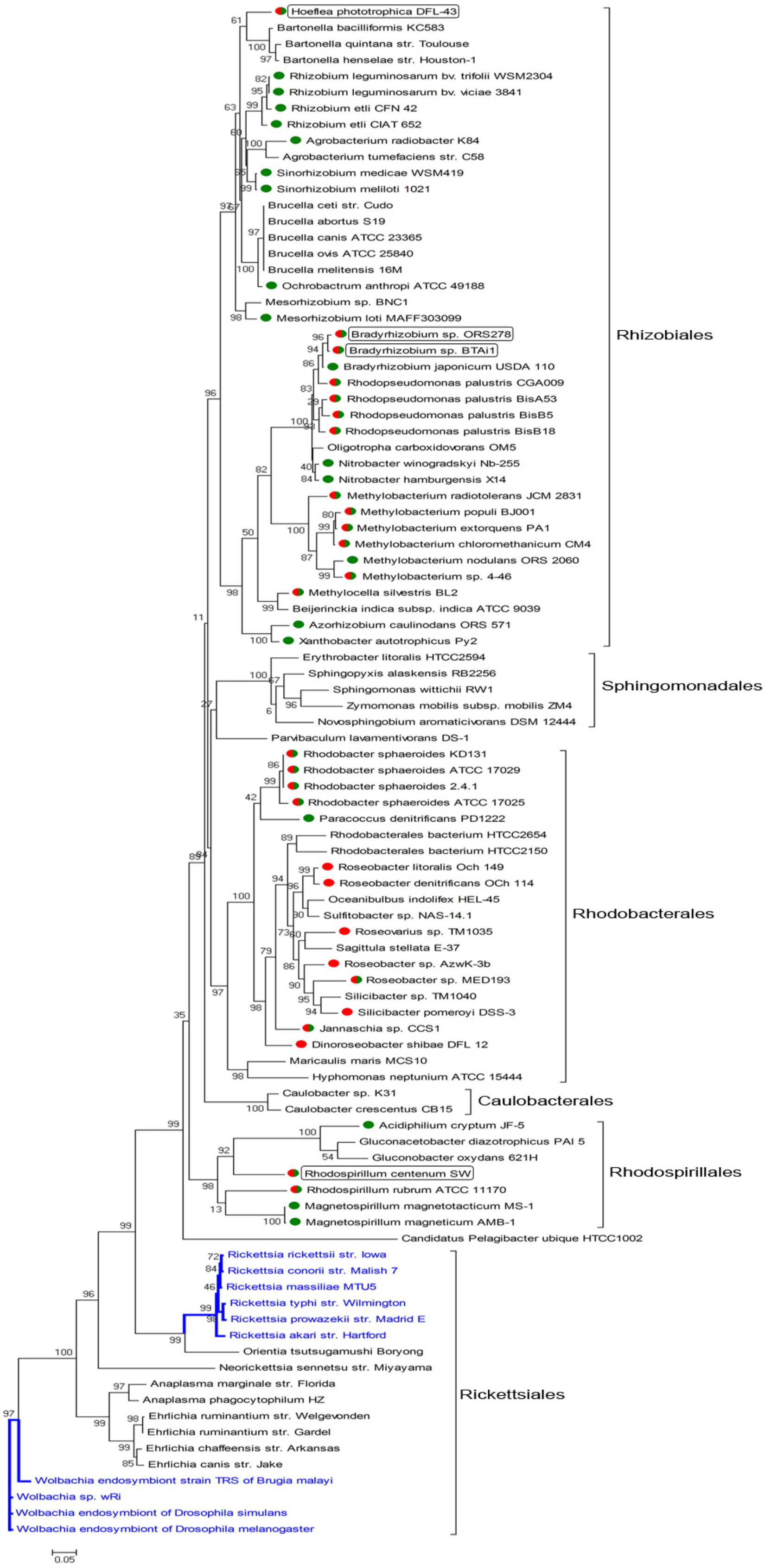

Figure S1. 16S rDNA phylogeny of the alpha-proteobacteria class indicating the distribution of Pk, Pdk, and PEPS. Rubisco-containing taxa are indicated with a green circle while phototrophic taxa are indicated with a red circle. Species that contain Rubisco and are phototrophic are indicated with half red, half green circle. All species contain Pk and Pdk except certain bacteria in the Rickettsiales order (blue), which possess Pdk only. Four bacteria possessing Pk, Pdk, and PEPS are boxed.

Table S1. Characterization of *R. centenum* flagella genes

Gene nomenclature	Copy number	Identity (%)*	Similarity (%)*	Predicted function
flgA	2	23	34	Flagellum basal body P-ring formation protein
flgB	2	43	60	Flagellum basal body protein
flgC	3	61(1:2), 31(1:3), 28(2:3)	77(1:2), 43(1:3), 44(2:3)	Flagellum basal body rod protein
flgD	2	23	35	Flagellum basal body rod modification protein
flgE	2	25	37	Flagellum hook protein
flgF	3	41(1:2), 3(1:3), 16(2:3)	60(1:2), 5(1:3), 28(2:3)	Flagellum basal body rod protein
flgG	2	61	78	Flagellum basal body rod protein
flgH	2	51	66	Flagellum L-ring protein
flgI	2	63	76	Flagellum P-ring protein
flgK	2	28	47	Flagellum hook-associated protein 1
flgL	2	20	35	Flagellum hook-associated protein 3
flhA	2	56	73	Flagellum biosynthesis protein
flhB	4	10(1:2), 49(1:3), 10(1:4), 9(2:3), 44(2:4), 10(3:4)	14(1:2), 67(1:3), 14(1:4), 13(2:3), 61(2:4), 15(3:4)	Flagellum biosynthesis protein
flhF	1	---	---	Flagellum GTP-binding protein
fliE	2	43	62	Flagellum hook-basal body complex protein
fliF	2	41	60	Flagellum M-ring protein
fliG	2	49	73	Flagellum motor switch protein
fliH	2	27	39	Flagellum assembly protein
fliI	2	59	73	H^+-transporting two-sector ATPase
fliK	2	21	27	Flagellum hook-length control protein
fliL	3	26(1:2), 21(1:3), 27(2:3)	34(1:2), 42(1:3), 42(2:3)	Flagellum basal body-associated protein
fliM	2	49	66	Flagellum motor switch protein
fliN	4	41(1:2), 35(1:3), 39(1:4), 40(2:3), 36(2:4), 29(3:4)	57(1:2), 54(1:3), 49(1:4), 62(2:3), 56(2:4), 48(3:4)	Flagellum motor switch protein
fliO	1	---	---	Flagellum assembly protein
fliP	2	48	65	Flagellum biosynthesis protein
fliQ	2	43	59	Flagellum biosynthesis protein
fliR	2	37	53	Flagellum biosynthesis protein
fliX	1	---	---	Flagellum assembly protein
flaA	1	---	---	Flagellin protein
flaB	2	27	41	Flagellin protein
flaF	2	35	49	Flagellin protein

flaG	1	---	---	Flagellin protein
flbD	1	---	---	Transcriptional regulatory protein
flbT	2	36	51	Flagellum biosynthesis repressor protein
fleN	1	---	---	Flagellum biosynthesis regulator

* The number indicates both identity and similarity resulted by each pairwise comparison if more than 2 copies of the gene are present.
